# Residual daytime sleepiness in patients with obstructive sleep apnoea syndrome: A service review in a tertiary sleep centre

**DOI:** 10.1007/s11325-026-03655-6

**Published:** 2026-03-28

**Authors:** Jasvinder Singh Kaler, Biswajit Chakrabarti, Sonya Craig, Joerg Steier

**Affiliations:** 1 Sleep Disorders Centre, Guy’s & St Thomas’ NHS Foundation Trust, London, UK; 2 Liverpool Sleep and Ventilation Centre, University Hospital Aintree, University Hospitals of Liverpool Group, Liverpool, UK; 3 Faculty of Life Sciences & Medicine, King’s College London, Centre for Human and Applied Physiological Sciences, London, UK

**Keywords:** CPAP, Epworth Sleepiness Scale, Stimulant Medication

## Abstract

**Rationale:**

Obstructive Sleep Apnoea Hypopnoea Syndrome (OSAHS) is frequently associated with excessive daytime sleepiness (EDS). There are sparse data on patients with OSAHS who are well treated with continuous positive airway pressures (CPAP) but continue to experience residual EDS. We sought to prospectively review a patient cohort to understand causes associated with this symptom.

**Methods:**

Patients with OSA and documented evidence of CPAP usage and respiratory control were recruited from a large tertiary referral centre (GSTT/2024/16137). Data are presented as mean (standard deviation).

**Results:**

Data were collected between 05/2024 and 01/2025. Out of 63 patients that were identified and pre-screened, we included 50 (age 56.7 (13.1) years, gender (male: female) 33:17, body mass index, BMI 36.6 (9.5) kg/m^2^). Patients had been diagnosed with OSAHS (AHI 36.7 (20.0) events x hour^− 1^; residual AHI on CPAP 4.2 (3.8) events x hour^− 1^; *p* < 0.0001), were adherent to CPAP (6.2 (1.6) hours x night^− 1^), and remained sleepy (pre-therapeutic ESS 17.6 (3.7) points; residual ESS on therapy 14.9 (4.0) points; *p* < 0.0001). Common comorbidities included chronic pain (46%), Restless Legs Syndrome/Periodic Limb Movement Disorder (34%), mental health issues (anxiety/depression, 68%), other sleep disorders (36%), cardiovascular disease (68%), and type-2 diabetes (32%). Co-medication included analgesia (22%), antidepressants (58%), dopaminergic drugs (10%); antihypertensives (56%), antidiabetics (22%), no patient was taking benzodiazepines.

**Conclusion:**

The identified cohort of patients with OSA and residual EDS still experienced a significant improvement of their symptoms with CPAP therapy. However, residual EDS may be attributable to substantial comorbidities with sleep fragmentation and co-medication.

## Introduction

Obstructive sleep apnoea (OSA), is a highly prevalent condition affecting around 1 billion people worldwide [1]. It is characterised by recurring pharyngeal collapse during sleep. Patients with OSA experience periods of apnoeas and hypopnoeas multiple times every hour, causing sleep fragmentation due to upper airway obstruction, high intrathoracic inspiratory pressure swings, intermittent hypoxaemia, sometimes hypercapnia, and arousal from sleep [2]. OSA is an independent risk factor for cardiovascular disease [2, 3] and treatment may improve long-term outcomes [4].

Patients with OSA typically present to the sleep centre with snoring, observed apnoeas, and associated daytime symptoms such as excessive daytime sleepiness (EDS), impaired cognition, low vigilance and limited concentration, with an impact on their quality of life [2, 5, 6]. These impairments can lead to significant societal and economic burden [7].

The primary target of any therapeutic approach for symptomatic OSA is to maintain and restore upper airway patency when asleep, normalise the breathing, avoid any sleep fragmentation, and, subsequently, alleviate symptom burden when awake [8, 9] Evidence-based treatment is available with continuous positive airway pressure, CPAP therapy, or the use of mandibular advancement devices, MAD [10–12]. Lifestyle advice, particularly focused on weight loss, avoidance of alcohol and smoking, regular sleep routine and posture is similarly encouraged.

Most patients improve symptomatically following normalisation of their breathing while asleep. Previous studies have shown that CPAP therapy improves sleepiness, as measured by the Epworth sleepiness scale (ESS), a subjective marker of sleepiness; a minimally clinically important difference (MCID) in the ESS is thought to be more than two points [13, 14].

However, about 15% of patients with OSA remain excessively sleepy despite appropriate airway treatment with good control of the respiratory events [15, 16], a phenomenon called residual excessive daytime sleepiness (rEDS). Data from the European Sleep Apnoea Database (ESADA) cohort indicate that up to 28.2% of patients with OSA, who receive treatment with CPAP, may still suffer from ongoing rEDS at follow up [17].

Despite rEDS affecting a significant proportion of patients on CPAP therapy, there remain sparse data on the prospective assessment of rEDS in patients with OSA who are well-controlled on CPAP therapy and who remain highly adherent to treatment. We sought to record prospective data in this cohort of patients, and to investigate the contributory factors of rEDS. We therefore reviewed patient who were under our sleep service and who remained excessively sleepy on treatment to better understand these aspects.

## Patients & methods

The study was registered with the clinical audit and service evaluation database (identifier: GSTT/2024/16137) in accordance with Trust Guidelines. The data acquisition period was between May and December 2024. Informed verbal consent was obtained by each participant during their outpatient clinic appointments.

Guy’s & St Thomas’ NHS Foundation Trust, London, has specialist clinics run by respiratory physicians with specialist interest in sleep, physiologists, technical support team, as well as senior clinical pharmacists, neurologists, psychiatrists, and neuropsychiatrists. There are established clinical pathways for weekly multidisciplinary team (MDT) pathways within the wider team, as well as monthly difficult-to-treat MDTs for patients with OSAHS. Patients with rEDS and optimally treated OSA were identified, selected, and offered to be referred to a pharmacist-led outpatient sleep clinic to consider eligibility of treatment with wake promoting agents.

Criteria required for patients to define rEDS:

i) Previous sleep study identifying OSAHS (apnoea-hypopnoea index, AHI > 15/hour),
ii) Epworth Sleepiness Scale (ESS) > 10/24 points at baseline,
iii) Established on CPAP therapy for a period of at least 3 months,
iv) Documented acceptable CPAP adherence (> 4 h usage x night^-1^; more than 70% of the nights),
v) CPAP therapy (Autoset CPAP, ResMed AirSense 10, Sydney/AUS; remote monitoring with Airview) was checked to adequately treat any component of sleep disordered breathing, i.e. Apnoea-Hypopnoea Index (AHI) of < 5 events x hour^− 1^ and no evidence of hypoventilation or significant oxygen desaturation in home-based sleep studies (watchPAT, ZOLL Itamar, Atlanta/GA, USA) on treatment,
vi) Patients in whom other metabolic, sleep or neurological/neuropsychiatric conditions, as well as prescribed medication associated with sedation/somnolence had been assessed and treated, as previously described [18],
vii) The effect of any concomitant medical conditions, prescribed medications, alcohol and stimulants (e.g., caffeine) taken by the individual that may contribute to EDS was considered and recorded.

An audit describing the services, pathways, and clinical outcomes (rEDS, as assessed by the Epworth Sleepiness Scale, ESS; Functional Outcome of Sleep Questionnaire, FOSQ; EQ-5D) was undertaken. No medications were prescribed during this project but it was envisaged that this work may inform the sleep centre’s area prescribing committees as to the possible numbers of eligible patients for wakefulness medication (‘stimulants’). We proposed that a pathway was drawn up regarding prescribing for rEDS based on information from this service evaluation and to potentially allow medication to be prescribed in the future for some of these patients.

The cohort of patients was fully described, as per above inclusion/eligibility criteria; the aim was to assess the work and flow of patients to identify a cohort of 50 such patients with rEDS who were adherent to CPAP therapy, and a selection of them in whom eligibility for adjunct therapy with wake promoting may be considered. The following variables were recorded: age, gender, ethnicity, body-mass-index (BMI), neck circumference, waist circumference, respiratory rate, blood pressure, heart rate, QTc time, blood panels (full blood count, renal function, liver function, iron panel, lipid profile, thyroid function), comorbidities, concurrent comedication, positive airway pressure (Autoset CPAP, ResMed AirSense 10, Sydney/AUS), mean usage per night (hours), mean duration since commencing treatment (years), adherence to CPAP (%), baseline and residual apnoea-hypopnoea-index (AHI, events per hour), baseline and residual oxygen desaturation index (ODI, 3% and 4%), baseline and follow up Epworth Sleepiness Scale (ESS), PROMIS sleep disturbance score, PROMIS sleep impairment score, EQ-5D visual analogue scale (VAS), hospital anxiety and depression scale (HADS).

### Questionnaires

The HADS consists of 14 questions (seven covering depression and seven covering anxiety). The maximum score for each subscale is 21. The questions may be scored from 0 to 3, with scores of at least 11 points indicating an abnormal result. The maximum score for each subscale is 21. The EQ-5D consists of two pages: the EQ-5D descriptive system and the EQ-5D visual analogue scale (VAS). The EQ-5D descriptive system comprises five dimensions: mobility, self-care, usual activities, pain and discomfort, and anxiety and depression. The five levels in each dimension are worded as (1) ‘not/no problems’, (2) ‘slight problems’, (3) ‘moderate problems’, (4) ‘severe problems’ and (5) ‘unable to’ (mobility, self-care, usual activities), ‘extreme’ (pain/depression), or ‘extremely’ (anxiety/depression). The VAS component of EQ-5D comprises a standard vertical 20-cm VAS that is calibrated from ‘the worst health you can imagine’ (scored 0) at its base to ‘the best health you can imagine’ (scored 100) at its apex. Respondents are asked to ‘mark an X on the scale to indicate how your health is today’. The PROMIS assessments are 8-item short form describing Sleep Disturbance and Sleep Impairment in individuals age 18 and older. Each item on the measure is rated on a 5-point scale (1 = never; 2 = rarely; 3 = sometimes; 4 = often; and 5 = always) with a range in score from 8 to 40 with higher scores indicating greater severity of sleep disturbance. The total raw score is converted to a T-score. The T-scores are interpreted with a minimum score of less than 55 meaning ‘None to slight disturbance/impairment’, and a maximum score 70 and over meaning ‘severe disturbance/impairment’.

### Joint MDT with sleep centre at NHS Aintree/Liverpool, UK

The same audit process took place in an independent sleep centre, NHS Aintree/Liverpool, UK and all pre-identified patients in both centres were discussed at a multidisciplinary team meeting (MDT, MS teams Microsoft, Seattle/WA, USA) following identification, and pre-assessment workup. Each patient’s case was presented to the institution’s team/together with another institution to discuss, comment, jointly learn from the cases, and harmonise decision-making across the involved teams; con-/discordance in therapeutic decisions was documented accordingly.

### Analysis plan

The following points were considered prior to data analysis:

- Consider pre-existing restless legs syndrome, major depression, narcolepsy and other concomitant hypersomnias,
- Descriptors of those with rEDS diagnosis including severity of the OSA,
- Estimate the frequency of rEDS in the selected cohort with no confounding issues.

Clinicians were to assess the relevance of confounders (e.g., well controlled hypothyroidism would not be relevant but undertreated hypothyroidism would etc.). Those considered suitable for potential medication would have their case discussed with the other centre to assess concordance on the suitability for the medication.

Confounders included shift work, alcohol intake, smoking, medications affecting sleep, medicines causing drowsiness, sleep hygiene, comorbidity, respiratory disease requiring long-term oxygen therapy, other medication and comorbidities, sleep hygiene.

### Statistical analysis

The Shapiro–Wilk test was used to test the normality of the data. Data are presented as mean and standard deviation, or interquartile range. For the quantitative variables, descriptive statistics were used. For selected variables 95% confidence intervals (CIs) were used. The ESS, AHI, ODI (all *n* = 50), and respiratory disturbance index (RDI) at baseline (polysomnography, *n* = 36; respiratory polygraphy, *n* = 14) and follow-up (home-based sleep study, watchPAT; *n* = 50) were compared by paired t-test for continuous variables. All statistical tests assessing levels of significance were two-sided and at a 5% level. Statistical analysis was performed on SPSS software (Version 30; IBM, NY/NY, USA). Patient data was anonymised and obtained from their respective electronic patient record EPIC (Hyperspace, 11.3.0.10; Verona/Wisconsin, USA) and recorded on a restricted access MS Excel spreadsheet (Microsoft v.1808; Seattle/Washington, USA).

### Data availability

Data may be made available following reasonable request and ethics/General Data Protection Regulation (GDPR) clearance.

## Results

Out of 63 screened patients (05–12/2024), we studied a cohort of 50 patients who were middle-aged, predominantly male, white, and obese (Table 1). Patients were not included if at any point they became ineligible, did not consent or could not be followed up. The majority of the patients included in the study had comorbid cardiovascular disease or mental health diagnosis. A notable proportion of patients were observed to have chronic pain (Table 2).

Antidepressants and antihypertensives were identified as the most frequently prescribed concurrent medications (Table 3). Three patients (6%) were observed to be taking concurrent melatonin. No patients included in the study was on prescribed benzodiazepines or Z-drugs. Vital observations during the assessment were largely within normal range indicating regular cardiovascular function while awake. However, respiratory rate was slightly increased and blood pressure was at the upper limit of normal, possibly indicating the exertional demand on the cardiovascular system when attending the outpatient clinic appointment face-to-face. The majority of the studied patients had some type of abnormal ECG (28/50, 56%), mostly with non-sinus rhythm (including atrial fibrillation, 22/50, 44%), brady- and tachycardia (2/50, 4%), or conduction block pictures (4/50, 8%, Table 4). The mean identified QTc time was identified to be within normal range, however, two patients (one male and one female) had prolonged QTc intervals at 457ms and 497ms, respectively.

The blood tests did not reveal any abnormal full blood count results, although the renal function in the studied cohort was slightly reduced, and the mean eGFR indicated stage 2 chronic kidney disease (CKD), as defined by the “Kidney Disease: Improving Global Outcomes” (KDIGO) working group [19]. One patient was observed to have stage 3a CKD, as defined by KDIGO. Iron studies were within normal range, and, notably, the mean ferritin was 84.4 µg x L− 1 and transferrin saturation (Tsat) was 24.4%. Thyroid function screening tests did not reveal any hypo- or hyperthyroidism (Table 5).

Patients had been diagnosed with confirmed and mostly severe OSA. They were established on CPAP therapy with good respiratory control several years earlier, and had documented high adherence to treatment (Table 6). The AHI, the ODI and the RDI, as measured by the home-based sleep study (watchPAT; ZOLL Itamar) on CPAP therapy were well-controlled and within normal range (Table 7). At the time of the assessment, patients indicated a degree of low mood, anxiety, and significantly reduced quality of life (QoL), with an impact on their QoL by sleep disturbance and sleep-related impairment (Table 8). The questionnaire data indicated a moderate degree of anxiety and depression, significant reduction in overall quality of life, and impact of sleep due to disturbance and impairment.

**Table 1:** Demographics of studied cohort (*n* = 50). Data are presented as mean (standard deviation), except for gender and ethnicity for which the count (n) and the ratio are provided

Demographics	
**Age (years)**	**56.7 (13.1)**
**Gender (male : female)**	**33 : 17 (1.9 : 1.0)**
**Weight (kg)**	**105.3 (25.4)**
**Body mass index**,** BMI (kg/m**^**2**^**)**	**36.6 (9.5)**
**Neck circumference (cm)**	**42.7 (4.8)**
**Waist (cm)**	**120.4 (22.6)**
**Ethnicity (white : Asian : black)**	**39 : 8 : 3**

**Table 2:** Identified pre-existing comorbidities in the study cohort. Data are presented as numbers, n (%)

Comorbidity	*n* (%)
**Cardiovascular disease**	**34 (68)**
**Mental health diagnosis (anxiety and depression)**	**34 (68)**
**Chronic pain**	**23 (46)**
**Restless Legs Syndrome/Periodic Limb Movement Disorder**	**17 (34)**
**Type-2 diabetes**	**16 (32)**

**Table 3:** Identified concurrent medications in the study cohort. Data are presented as n (%)

Concurrent medication	
**Antidepressants**	**29 (58%)**
**Antihypertensives**	**28 (56%)**
**Analgesia**	**11 (22%)**
**Antidiabetics**	**11 (22%)**
**Dopaminergic drugs**	**5 (10%)**

**Table 4:** Vital observations when attending the outpatient clinic assessment (face-to-face). Data are presented as mean (standard deviation). ECG QTc, corrected QT time in the ECG recording

Observations	
**Heart rate** (minute^− 1^)	**75.3 (11.4)**
**Respiratory rate** (minute^− 1^)	**17.7 (1.7)**
**Blood pressure**,** systolic (mmHg)**	**131.4 (17.5)**
**Blood pressure**,** diastolic (mmHg)**	**79.7 (11.3)**
**SpO**_**2**_ **(%)**	**95.2 (2.5)**
**ECG QTc (ms)**	**420.3 (29.5)**

**Table 5:** Blood test results at attendance in the outpatient clinic. Normal ranges indicated in italics, based on the Hospital Trust’s laboratory values. eGFR= Estimated glomerular filtration rate; ALT= Alanine transaminase; Tsat= Transferrin saturation; TC= Total cholesterol; LDL = low density lipoprotein; TSH= Thyroid stimulating hormone

Blood Test Results	
**Haemoglobin** (g x L^− 1^) (125–170)	**139.9 (14.6)**
**eGFR** (mls x min^− 1^)	** 74.2 (38.0)**
**ALT (U x L**^**− 1**^**)** ***(≤ 55)***	**32.3 (19.7)**
**Ferritin (µg x L**^**− 1**^**)** ***(15–200)***	**84.4 (56.9)**
**Tsat (%)** ***(20–45)***	**24.4 (15.7)**
**Iron (µmol x L**^**− 1**^**)** ***(11.6–31.3)***	**16.3 (6.7)**
**TC (mmol x L**^**− 1**^**)** ***(< 5.0)***	**4.5 (1.3)**
**LDL (mmol x L**^**− 1**^**)** ***(< 3.0)***	**2.4 (1.0)**
**TSH (mIU x L**^**− 1**^**)** ***(0.35–4.94)***	**1.8 (1.1)**

**Table 6:** Data indicating pressure, usage and adherence to the CPAP therapy (ResMed, AirSense 10), presented as mean (standard deviation)

Continuous Positive Airway Pressure, CPAP Therapy	
**CPAP level (cmH**_**2**_ **O)**	12.8 (4.0)
**CPAP usage (hours x night** ^**− 1**^ **)**	6.2 (1.6)
**CPAP adherence (% of nights used)**	87.0 (13.7)
**CPAP treatment duration (years)**	3.2 (2.0)

**Table 7:** Respiratory indices of the patients with OSA improved sufficiently and were within normal range on CPAP therapy. Sleepiness, as measured by the ESS, also indicated significant improvement with CPAP therapy, but remained elevated due to starting off from a high baseline value. 95% confidence intervals for delta in parenthesis

Obstructive Sleep Apnoea				
	Baseline	Follow up	Delta	*p*-value
**Apnoea-Hypopnoea-Index**,** AHI (hour**^**− 1**^**)**	36.7 (20.0)	4.2 (3.8)	−32.6 (−38.5; −26.8)	< 0.001
**3% Oxygen Desaturation Index**,** ODI (hour**^**− 1**^**)**	21.6 (21.2)	3.8 (3.1)	−17.6 (−24.5; −10.7)	< 0.001
**Respiratory Disturbance Index**,** RDI (hour**^**− 1**^**)**	38.0 (19.9)	4.4 (4.0)	−33.6 (−40.0; −27.2)	< 0.001
**Epworth Sleepiness Scale**,** ESS (out of 24 points)**	17.6 (3.7)	14.9 (4.0)	−2.7 (−3.4; −2.0)	< 0.001

**Table 8:** HADS, Hospital Anxiety and Depression Scale; EQ-5D, European Quality of Life 5-Dimensions questionnaire; PROMIS, Patient-related Outcome Measurements

Questionnaires	
**HADS, Anxiety (out of 21 points)**	**11.9 (5.1)**
**HADS, Depression (out of 21 points)**	**11.8 (5.8)**
**EQ-5D (out of 100 points)**	**44.8 (23.2)**
**PROMIS**,** Sleep Disturbance (T-score)**	**61.5 (8.9)**
**PROMIS**,** Sleep-related Impairment (T-score)**	**64.2 (12.4)**

There were no significant correlations between the ESS at baseline, follow up, or the delta ESS and any of the other demographic and sleep related variables (p = n.s.). A linear regression analysis with residual ESS as dependent variable was performed (enter, stepwise) including age, gender, BMI, baseline AHI, baseline ESS, HADS-A and HADS-D, as well as CPAP adherence, but did not reach any level of significance (p = n.s.).

## Discussion

In a select cohort of patients with severe OSA and good respiratory control on Autoset CPAP therapy, with proven evidence of high therapeutic usage using remote monitoring, and with good respiratory control, as confirmed by a home-based sleep study (watchPAT), residual excessive daytime sleepiness, as defined by the ESS, frequently remains an issue due to comorbidities and comedication. However, despite the level of residual sleepiness the symptom still improved significantly with treatment, even in those who remain above a threshold typically associated with severe sleepiness (i.e., ESS > 10 points). In our cohort, the ESS improved by between 2 and 3 points with CPAP therapy. It is important that the cohort of patients studied were obese, had significant comorbidities and comedication that could have impacted on the residual symptoms observed. Of specific interest are cardiovascular conditions, such as arrhythmias (e.g., atrial fibrillation), mental health issues (e.g., anxiety and depression), and sleep fragmenting conditions (e.g., chronic pain, periodic limb movement disorder). The symptomatic burden experienced by this cohort of patients is further confounded by their medication, particularly antidepressants and analgesia which, in themselves, may contribute to sleepiness due to their side effect spectrum.

### Clinical significance

A general understanding of the interaction between OSA severity, different phenotypical presentations, the influence of different genders and clinical symptoms is essential for the development of personalised treatment strategies for patients with OSAHS.

However, it is difficult to define the prevalence of residual excessive daytime sleepiness in patients with OSAHS accurately out of several reasons. Efficacy of primary airway treatment needs to be established over sufficient time, with no residual apnoeas and hypopnoeas, and good therapeutic adherence. Additionally, it is relevant whether patients have pre-existing comorbidities, for example idiopathic hypersomnolence or central hypersomnias of other origin [9]. However, previous studies estimated the prevalence of rEDS in patients with OSA to be between 11–18% [20], while the ESADA data estimated it to be up to 25% [17]. I ccccg, we enrolled selected patients with pre-defined criteria to record their symptoms, comorbidities and comedication during follow up. It is therefore important to highlight that patients who remain affected by residual excessive sleepiness also have the potential to experience a significant improvement in their symptoms, which is within the expected range of the MCID for ESS with CPAP therapy in OSAHS [13, 14]. Therefore, it is reasonable to describe rEDS as a ‘threshold effect’, as higher or lower cutoffs lead to a shifting of the rates of patients with OSAHS affected by this category. It also deserves outlining that even those patients who remain excessively sleepy should be encouraged to continue using CPAP therapy, as it is likely they would be more symptomatic without treatment.

Importantly, there are several potentially modifiable co-factors in addition to the provision of optimal respiratory care:

A) Comorbidities were frequently associated with factors that could exacerbate chronic sleepiness. Chronic pain in particular was a frequent confounder in our cohort, it impacts on sleep and results in spending less time in slow wave and REM sleep. Alpha-intrusion in stage N3 may lead to frequent shifts to N2 (cyclic alternating pattern) and microarousals, periodic limb movements, sweating and palpitations result in less REM sleep. Patients may experience this as increased wake-after-sleep-onset (WASO) time and sleep maintenance insomnia [21]. Treatment of anxiety and depression, as well as sleep fragmenting disorders (e.g., RLS/PLMD) may also be optimised to improve symptom relief.
B) Comedication included analgesia, antidepressants, and antihypertensives. These medications should be reviewed to avoid sedative and/or fatiguing properties and direct effects on sleep interference. The indication to use medication to tackle underlying comorbidities should not stop prescription of effective treatment. However, if alternative medications within these major drug classes are clinically suitable then a switch should be considered to reduce contributing further to EDS. For analgesia, non-steroidal anti-inflammatory drugs (NSAIDs) are preferable to opioids, gabapentinoids, antidepressants, or antiepileptics. For depression, SSRIs and SNRIs are preferable to TCAs, MAO inhibitors and atypical antidepressants which have sedative properties [22, 23]. From a cardiovascular pharmacological aspect, hydrophilic and lipophilic β-Blockers may be associated with adverse fatigue and sedation. A meta-analysis of 55 studies has previously shown that approximately 24% of patients who take atenolol reported sedation when compared to placebo [24]. Another meta-analysis found a significant increase of fatigue in patients taking β-blockers [25].
C) Obesity needs to be highlighted as well, as it is not only a common comorbidity found in the general cohort of patients with OSAHS, but is also independently associated with excessive daytime sleepiness [26]. Furthermore, effective weight loss services can help to improve this confounder and, potentially, even reverse the underlying pathophysiology of OSAHS (e.g., weight loss drugs, bariatric interventions) [27].

Deducting from the above information, it is essential to prepare a holistic and coordinated approach when reviewing patients with OSAHS. Traditionally, the respiratory physician will have taken care of patients with OSAHS. However, with rising age of the general population, more frequent comorbidities, widely available comedication, and, possibly, metabolic factors need to be considered when patients continue to experience symtpoms, and this requires more resources. A synchronised approach between primary care physician (General Practitioner), pharmacist, the diabetic team and sleep-centre based physician would be ideal to improve clinical outcomes in these patients. It is, unfortunately, understandable that this would also require more healthcare resources with spiralling costs. However, we should expect direct communication and discussion between the different professions involved to improve patient benefit, while interdisciplinary team conferences may also offer the chance to discuss selected and more complex cases in more detail [28].

Finally, stimulant medication is licensed in some countries to treat patients with OSAHS and residual sleepiness (e.g., Modafinil, Pitolisant, Solriamfetol) [18], while sometimes off-label use of stimulant medication may have also been trialled in the past (e.g., Methylphenidate, Dexamphetamine). However, other healthcare systems may be more restrictive in the use of stimulant medication and advise against the use of medication in the context of OSAHS-related symptoms, while still permitting them for symptom control in patients with central hypersomnias. The pro’s and con’s of symptom relief by medication should be carefully considered prior to commencing any patient, potentially for the rest of their lives, on drug therapy [9, 18, 29, 30].

### Limitations to the Study

Although this study followed a prospective study design of a clinically-pragmatic service evaluation to assess and characterise patients with residual excessive sleepiness, there are relevant limitations to our findings. Firstly, this was not a controlled study with comparison group and any clinically significance of our observations, as outlined above, should be validated in prospective controlled trials. However, this may prove to be impossible due to the complexity and the interaction of the many confounders involved (i.e., comorbidities, comedication, obesity). Secondly, it needs to be highlighted that even in our cohort of residually sleepy patients we observed a significant and clinically meaningful improvement in the level of sleepiness with CPAP treatment. This raises the question to what extent the ESS, particularly a threshold to define excessive daytime sleepiness (e.g., > 10 points), shold be used as a reliable marker for residual symptoms. Objective measurements, such as the mean sleep latency, or other subjective markers of sleepiness, for example the Stanford Sleepiness Scale, may need to be considered to understand the full picture. Sleepiness is a multi-dimensional and complex symptom, which may be influenced by other factors that may include anxiety, depression, fatigue, and mastery of the condition [31]. In the absence of these data, it remains advisable to consider both a relative improvement in the symptom as well as an absolute threshold score of the ESS. Thirdly, our data describe a detailed service review, but only contained a relatively small number of patients. The limited sample size, particularly the low number of females and the diversity of ethnicities, restricts the ability to compare within these specific groups. It would be important to seek verification of our findings from larger datasets, such as form the European Sleep Apnoea Database (ESADA) cohort [17]. Fourthly, we agree that the polysomnographical recording of a sleep study provides more information on the sleep physiology with relevant insights and explanations into the aetiology of residual sleepiness. In our study, most of the patients (*n* = 36) had initially been assessed with polysomnographies, but a minority (*n* = 14) underwent initial inpatient respiratory polygraphies. This distinction is relevant to the interpretation of residual sleepiness, as polysomnography results provide information on sleep architecture, arousals, and comorbid sleep-fragmenting disorders (e.g., periodic limb movement disorder or insomnia-related fragmentation) that cannot be assessed using polygraphy alone. Finally, we accept that nocturnal hypoventilation, as defined by hypercapnic episodes, should be ruled out; however, this requires the measurement of arterial blood gases, transcutaneous capnography, or other surrogate markers. Unfortunately, these assessments are not standard in the assessment of OSAHS patient in the sleep centres in the NHS. We do have, however, reassuring repeat home-based sleep studies in this cohort of patients (watchPAT) and data from remote monitoring using Autoset CPAP devices (ResMed). These data confirm sufficient control of the OSA, making it unlikely that the studied cohort would have been significantly affected by substantial hypoventilation associated with obesity, but it cannot entirely be excluded that in some of the patients with OSA marginally elevated levels of CO_2_ could have been present.

## Conclusion

Patients with OSA on CPAP therapy who remain severely affected by daytime sleepiness require a thorough and holistic assessment to better understand and treat their comorbidities, optimise their medication to avoid sedative side effects, and in collaboration with the General Practitioner (and, possibly, other professionals such as pharmacists) manage additional factors (e.g., obesity) to improve symptom burden and quality of life. Such an approach requires additional healthcare resources in highly-frequented clinical sleep services, but has the potential to lead to tangible improvements in symptom control and quality of life of patients with otherwise disabling levels of sleepiness.
